# Supplementary material for: High-fidelity 3D live-cell nanoscopy through data-driven enhanced super-resolution radial fluctuation
Source: Nat Methods. 2023 Nov 13;20(12):1949–56. doi: 10.1038/s41592-023-02057-w (PMC10703683; doi:10.1038/s41592-023-02057-w)
Supplement: Supplementary file 1 — Supplementary Notes 1–3, Tables 1–5, Figs. 1–7 and Videos 1–5. [file 41592_2023_2057_MOESM1_ESM.pdf]

nature methods

Article https://doi.org/10.1038/s41592-023-02057-w

High-fidelity 3D live-cell nanoscopy through data-driven enhanced super-resolution radial fluctuation

In the format provided by the authors and unedited

Supplementary information table-of-contents:

Supplementary Note 1-3
Supplementary Table 1-5
Supplementary Fig. 1-7
Supplementary Movie 1-5
References

Supplementary Notes

Supplementary Note 1: (e)SRRF and its artifacts

The presence of artifacts in the SRRF reconstruction have been observed previously[1]. Here, we highlight an instance where they appear quite clearly (Supplementary Fig. 2). We generated a simulated dataset where the underlying fluorophore arrangement follows a fan configuration. Supplementary Fig. 2 shows the reconstructions obtained from SRRF and eSRRF, as well as quantitative measures of image quality based on our SQUIRREL method[2]. We observe a clear visual and quantitative improvement of the method. Both resolution-scaled Pearson coefficient (RSP) and resolution-scaled error (RSE) show values associated with improved quality.

Supplementary Note 2: eSRRF processing and analysis concepts

The eSRRF approach reconsiders the concepts presented in SRRF and uses knowledge of the PSF and the imaging set up to enhance the reconstruction. The method consists in a 2-step process (see Fig. 1): a spatial analysis performed on each image of the temporal stack, creating the Radial Gradient Convergence (RGC) stack followed by a temporal analysis on the RGC stack.

Supplementary Note 2.1: Spatial transformation: the Radial Gradient Convergence transform

The SRRF concept exploits the radial symmetry of the signal in the image obtained from a discrete number of emitters in the image. This analysis requires the calculation of image gradients in every single pixel of the image[3]. Additionally, the super-resolved image needs to be reconstructed on a finer pixel grid than the acquisition. Therefore, the first step that we take in the spatial reconstruction is to spatially interpolate the raw image by a factor set by the user-set *magnification* parameter. This is done using Fourier Interpolation via Discrete Hartley Transform using JTransforms 3.1, https://github.com/wendykierp/JTransforms. The data is mirror-padded to represent a 2^nx2^n 2D data block prior to the FHT and the interpolated image is cropped back down to the original image width and height after the interpolation. We have shown that FHT

interpolation at an early step of the analysis reduces the occurrence of macro-pixel patterning (Extended Data Fig. 1). A set of interpolated frames are sent to the GPU defined automatically by the reconstruction settings. Subsequent calculations are performed on the GPU.

The vertical and horizontal image intensity gradients are then calculated from the interpolated images using the Roberts cross method[4].

$$G_a(i+0.5, j+0.5) = I(i,j) - I(i+1, j+1)$$
$$G_b(i+0.5, j+0.5) = I(i,j+1) - I(i+1, j)$$

Where G_a and G_b represent the 45-degree angle intensity gradients in the interpolated image. By using Robert's cross, the gradients calculated correspond to those in the corner of each pixel in the original image. These calculations are performed in continuous space based on a Catmull-Rom interpolation[5].
The gradients are then rotated by 45 degrees again to be in line with the vertical and horizontal axes of the image using standard 45 degrees rotation matrix calculation, so as to obtain G_x and G_y in each pixel (*i,j*). We found that, compared to other methods, Robert's cross generally gives the best estimates of local gradients.

Then the RGC transform is calculated for every pixel of the image. The user-input *Radius R* in pixels represents the FWHM of the expected PSF, thus, the PSF standard deviation can be estimated as follows:

$$\sigma = \frac{Radius}{2.355}$$

σ can be used to calculate the convergence weighting factor *W* as well as the size of the relevant local area over which to calculate the RGC.
For a particular pixel of interest, the RGC is calculated by summing the weighted gradient convergence (D_k) from all the surrounding pixels in a disk of radius 2σ+1, called Δ. This radius of calculation allows to speed up the process by only using the relevant local information to the pixel of interest and was determined empirically.
The RGC in the pixel (i_0,j_0) is computed as follows:

$$RGC(i_0, j_0) = \sum_{i,j}^{\Delta} W(i_0, j_0, i, j) \times D_k(i_0, j_0, i, j)$$

For each pixel in Δ, the distance *d* to the pixel of interest is computed and used to calculate the weighting factor *W*:

$$d = \sqrt{(i-i_0)^2 + (j-j_0)^2}$$

and

$$W(i_0, j_0, i, j) = W(d) = \left[d \times e^{-\frac{d^2}{2\sigma^2}}\right]^4$$

Which is based on the 4-th power of the derivative of a Gaussian pattern. Using the derivative of the Gaussian pattern allows us to weigh more highly the pixels where a strong gradient would be

expected if a molecule was present in the pixel of interest. The 4-th power was empirically derived to provide the best local gradient sensitivity.

The dot product of the gradient vector in the adjacent pixel and the distance vector between the adjacent and pixel of interest is computed to know the orientation of the gradient vector with respect to the pixel of interest. If the gradient vector points toward the pixel of interest, the distance of the tangent is found by taking the cross product of the distance vector and the gradient vectors. This is similar to what was done before with SRRF. This is used to calculate the gradient convergence for a particular location pair:

$$D_k(i_0, j_0, i, j) = 1 - \frac{\left|G_y(i,j) \times (i - i_0) - G_x(i,j) \times (j - j_0)\right|}{d\sqrt{G_x(i,j)^2 + G_y(i,j)^2}}$$

This essentially computes the smallest distance between the gradient vector in (*i,j*) and the point of interest (i_0,j_0), normalized by the distance between these 2 points. D_k then becomes 1 if the gradient points exactly at (i_0,j_0), and decreases as the vector points further and further away.

NB: the RGC grid and the gradient grid are effectively on the same sized grid but the gradients are computed on the corner of the pixels whereas the RGC (and interpolated intensities) are computed on the centre of the pixels.

In the last step, to fine-tune the PSF sharpening power, a contrast enhancement of the RGC is performed by applying the user defined Sensitivity *S* value as a power to the RGC map.

Supplementary Note 2.2: Temporal transformation: temporal cross-correlation analysis

eSRRF provides three different temporal transformation strategies that each perform best in different emitter density and fluctuation regimes (see Extended Data Fig. 4). A temporal average projection (*AVG)* of the RGC map for each pixel (i_0,j_0) provides robust results with low sensitivity to noise, over a wide range of emitter densities.

$$AVG\ (i_0, j_0) = \langle\ RGC_t(i_0, j_0)\ \rangle,$$

$\langle \dots \rangle$ indicates the average over time. However, an additional resolution improvement can only be achieved by higher-order temporal correlations. The temporal variance projection (*VAR*) which corresponds to cross-cumulant of 1st order without temporal offset and the 2nd order temporal auto-cumulant (*TAC2*) which corresponds to the cross-cumulant of 1st order with offset of 1 frame[5] provide an additional resolution gain with significant improvements in fidelity and contrast by analysing the temporal fluctuations:

$$VAR\ (i_0, j_o) = \langle \delta RGC_t\ (i_0, j_0) \cdot \delta RGC_t(i_0, j_0)\ \rangle,$$

$$TAC2\ (i_0, j_0) = \langle \delta RGC_t\ (i_0, j_0) \cdot \delta RGC_{t+\delta t}\ (i_0, j_0) \rangle,$$

with $\delta RGC_t\ (i_0, j_0) = RGC_t(i_0, j_0) - \langle RGC_t(i_0, j_0) \rangle$.

From an implementation point of view, the GPU calculates both the temporal average of the RGC maps and the temporal average of the square of the RGC maps. The final VAR is then computed on CPU as follows:

$$VAR(i_0, j_0) = \sqrt{\langle\, {RGC^2}_t(i_0, j_0) \ \rangle - \langle\, RGC_t(i_0, j_0) \ \rangle^2}$$

A similar approach is taken for *TAC2*.

Supplementary Note 3: 3D eSRRF using MFM

Supplementary Note 3.1: General concepts and implementations of 3D eSRRF using MFM

(e)SRRF can be extended to provide 3D super-resolution, with resolution improvement in both lateral and axial directions, as long as the fluctuations of emitters can be captured in 3D. This can in principle be achieved by any imaging system capable of imaging in 3D with a speed sufficiently higher than the time scale of the fluctuations of the emitters. However, the most robust way to ensure this condition is to capture multiple planes in the axial direction simultaneously. MFM[6–8] offers a robust method for simultaneous acquisition of multiple planes across a large volume, while retaining diffraction-limited resolution throughout the volume. Briefly, MFM uses a specialized optical assembly in the detection path of a widefield microscope system. MFM allows for different planes in the sample volume to be focused on different parts of the camera plane. A multifocus grating splits the emission into a desired distribution of the diffraction orders while adding a different defocus to each. A multifaceted blazed grating followed by a multifaceted prism corrects any chromatic dispersion introduced by the first grating and repositions the images onto the camera.

In particular, the MFM microscopy system used in this study was made so that we could measure emitter fluctuations over a whole volume using Nyquist sampling of the PSF in both lateral and axial directions. This readily allows eSRRF to extend the reconstruction in the third dimension, as in this condition, ignoring the different spatial sampling in lateral and axial directions, we can consider that emitters lead to not axially elongated but spherical patterns in 3D, further simplifying the extension of the RGC to the axial direction.
Therefore, the gradients, convergence weighting factor *W* and weighted gradient convergence described in Supplementary Note 2 can be simply extended to 3D. Once the RGC can be estimated for the whole volume acquired by MFM, the simultaneous acquisition of all planes can be extended to a time series of a few hundred frames (in the same way as the 2D acquisition), and the temporal analysis described in Supplementary Note 2 can also be used.
This leads to a full 3D reconstruction applying the eSRRF method from a typical MFM time series acquisition, without any modifications of the optical MFM set up. We also expect that it may be possible to perform 3D eSRRF using standard multi-plane imaging system that simply shift the detection plane axially in the detection arm of the microscopy system, accepting a loss of resolution due to spherical aberrations, but with some minor adaptation of the 3D eSRRF

algorithms taking into account the widening of the fluctuation patterns in space as we move away from the plane of best focus. In practice, this can be achieved by gradually adapting the *Radius* parameter across the multiple planes. The advantage of MFM is that this is not necessary.

Supplementary Note 3.2: MFM and 3D eSRRF registration procedure

In MFM, all the different axial planes are obtained simultaneously on the same camera chip but at different positions. For instance, in the setup used in this study, 9 different planes are acquired with ~400nm axial steps between them, covering ~3.6um depth into the sample.
However, in order to perform eSRRF in 3D (or to do any analysis or visualization in the volume), it is necessary to know how the different parts of the camera register to each other in 3D: this is the step of 3D registration.
The way the diffraction grating is made determines the relationship between where each plane is on the camera and where it is in the sample. This relationship is fixed. Their actual registration at the nanoscale is also defined by the optical alignment and imperfections in the optical assembly. Therefore, we implemented an analytical procedure to extract the nanoscale registration of each plane in 3D similarly to what was previously reported in Hajj et al PNAS 2014 and provided the tool for it as part of the NanoJ-eSRRF package (*3D eSRRF - Get spatial registration from MFM data*).
To extract the 3D registration from the data, it is necessary to provide an axial scan through a sample of defined axial size, such as a layer of fluorescent beads on a coverslip or embedded in a hydrogel. From this stack, it is possible to recover the position of the same structure in 3D throughout all the measured planes of the MFM system. This makes it possible for the algorithm to spatially register the data in the lateral direction, using the same routines as the Drift correction implemented as part of the NanoJ-Core package[9]. Additionally, the axial position of the identical plane through the axial stack allows us to extract the axial offset between consecutive planes, similarly with sub-axial step accuracy.
When the registration routine is run on these data, it gives back the 3D registration table for the imaging setup and spectral channel in question. This table can be used to convert any MFM data acquired in the same optical setup to a 3D stack using the *3D eSRRF - Apply spatial registration to MFM data* available in the NanoJ-eSRRF package. This routine uses bicubic interpolation to register the images with the extracted registration parameters. This allows the visualization of any 3D data with renderers like ClearVolume[10] or others.
But for (e)SRRF, it is more accurate to use the registration parameters directly in the routine, avoiding the step of bicubic interpolation performed during the above registration. 3D eSRRF uses the registration parameters to adjust the location of the different points used to calculate the gradients and the weighting to automatically align all the data in 3D.

Supplementary Tables

Method	Reference	Basic principles	Implementation	GPU acceleration	Image quality and resolution validated	3D	In depth
eSRRF	this paper	Radiality and temporal cross-correlation	Fiji	Yes	Yes	Yes	Yes
SRRF	Gustafsson *et al., 2016* [11]	Radiality and temporal cross-correlation	Fiji	Yes	No	No	Yes
MUSICAL	Agarwal *et al., 2016*[12]	Multiple signal classification	Fiji	Yes	No	No	Yes
3B	Cox et al., 2011[13]	Bayesian analysis of blinking and bleaching	Fiji	Yes	No	Yes[14]	Yes
SOFI	Dertinger *et al., 2009*[15]	Temporal cross-correlation	MATLAB	No	No	Yes	Yes
SACD	Zhao *et al., 2020*[16]	Deconvolution, temporal cross-correlation	MATLAB	No	No*	Yes[17]	N/A

Supplementary Table 1: Comparison of fluctuation-based super-resolution microscopy methods. *FRC resolution estimate based optimization

Method	Reference	Basic principles	Implementation	Artifact reduction	Resolution enhancement	Image quality and resolution validated	3D	Live-cell compatible
eSRRF	this paper	Radiality and temporal auto-correlation	Fiji	Yes	Yes	Yes	Yes	Yes
CERN	Zeng *et al., 2020*[18]	cross-cumulant analysis and SRRF	MATLAB & Fiji	Yes	Yes	No	No	Yes
JT-SRRF	Zeng *et al., 2018*[19]	Emitter density reduction by joint tagging with spectrally separated QDs	Sample preparation	No	Yes	No	No	No
gmSRRF	Gong *et al.* 2021[20]	Gradient variance modified SRRF	MATLAB	Yes	Yes	No	No	Yes
(Ex-) FEAST	Wang *et al.,* 2020[20]	Airyscan imaging with SRRF processing (of expanded samples)	Airyscan imaging (and expansion microscopy)	No	Yes	No	No	Yes (not for QD/Ex)
ONE	Shaib *et al., 2022*[21]	Confocal or WF imaging of 10x expanded samples and SRRF processing	Fiji	No	Yes	No	No	No
ExSRRF	Kylies *et al.*, 2023[22]	WF imaging of 4x expanded tissue samples and SRRF processing	LED-based WF microscopy and expansion microscopy	No	Yes	No	No	No

Supplementary Table 2: Recent adaptations and extensions of SRRF. Abbreviations: CERN - cross-cumulant enhanced radiality nanoscopy, JT - joint tagging, QD - Quantum dot, gmSRRF - gradient variance modified SRRF, (Ex-)FEAST - fluctuation-enhanced Airyscan technology (and sample expansion microscopy), ONE - one nanometer expansion microscopy

**Parameter**	**Description**
Magnification *M*	Define how the camera pixels are split into sub-pixels for the RGC estimation.
Radius *R*	Define the receptive field size that is used to calculate the RGC, the size should represent the FWHM of the expected PSF.
Sensitivity *S*	Define the sensitivity factor to fine-tune the PSF sharpening power applied by the RGC.
Number of frames for eSRRF	Define the size of the frame window for the temporal analysis.
Vibration correction	Activate vibration correction based on cross correlation.
Temporal analysis method	Select AVG, VAR and/or TAC2 as a temporal analysis method.
Rolling analysis	Activate rolling analysis and define frame gap size to increase temporal sampling by interleaved reconstruction.
3D eSRRF	Activate 3D eSRRF analysis and define offset between axial planes in nm.

Supplementary Table 3: eSRRF parameters

**Dataset**	**Figure panel**	**Laser intensity**	**Imaging speed/time**	**Analysis parameters**	**Resolution estimate** mean ± standard deviation
DNA-PAINT of microtubule network in fixed COS-7 cells	Graphical Abstract a), 1, S1, ED4, S3	1 kW/cm^2	33 Hz/25 min	eSRRF: M=5, R=0.5, S=1, VAR, all frames<br>SRRF: M=5, R=0.5, A=6	WF:<br>FRC = 215 ± 20 nm<br>Decorrelation = 424 nm<br>eSRRF:<br>FRC = 84±11 nm,<br>Decorrelation = 129 nm<br>SRRF:<br>FRC = 112±40 nm,<br>Decorrelation = 131 nm<br>SMLM<br>FRC = 71 ± 2 nm,<br>Decorrelation = 82 nm
COS-7 cells expressing Lyn kinase – SkylanS[23]	2	39 W/cm^2	33 Hz/15s	M=4, R=1.5, S=4, VAR, 200 frames	-
Live-cell LLS of the ER in Jurkat cells	3b-c), M2	n/a	100 Hz/130 s	M=5, R=3.5, S=2, AVG, 100 frames	WF:<br>FRC = 164±9<br>Decorrelation = 455 nm<br>eSRRF:<br>FRC = 84±43 nm<br>Decorrelation = 207 nm
Live-cell HiLO-TIRF microscopy of ER in COS-7	3d), ED1, ED7, M3	123 mW/cm^2	95 Hz/60 s	M=5, R=2, S=1, AVG,100 fr, rolling analysis gap=10 frames	WF:<br>FRC = 254±11 nm<br>Decorrelation = 637 nm<br>eSRRF:<br>FRC = 143±56 nm,<br>Decorrelation = 255 nm

Live-cell MFM of mitochondria network in HeLa cells (set1/set2)	4, ED10/M5	21.4 W/cm^2/11.5 W/cm^2*	50 Hz/20 s-3min20s	M=4, R=2, S=1, AVG, 100 frames, rolling analysis gap=25 frames	WF:<br>FRC(xy) = 231 ± 10 nm/317 ± 22 nm<br>Decorrelation(xy) = 481 nm/486 nm<br>FRC(xz)=-/-<br>Decorrelation(xz) = 490 nm/483 nm<br>eSRRF:<br>FRC(x,y) = 74 ± 12 nm/124±60 nm<br>Decorrelation(xy) = 184 nm/239 nm<br>FRC(xz) = 173 ± 19 nm/222 ± 26 nm<br>Decorrelation(xz) = 236 nm/285 nm<br>Deconvolved:<br>FRC(x,y) = 197.0±7.1 nm/146 ±58 nm<br>Decorrelation(xy) = 285 nm/272 nm<br>FRC(xz) = 297±89 nm/244±20 nm<br>Decorrelation(xz) = 421 nm/300 nm
ARGO-SIM calibration slide	ED2, ED6	-	86 Hz/1min	eSRRF: M=2, R=1, S=2, AVG, 5000 fr<br>SRRF: M=2, R=0.6, A=2, TRPPM, 5000fr	-
Life-cell SIM imaging of cultured neurons expressing Tubulin-eGFP	ED5a)	488 nm: 15.1 W/cm^2	5 Hz, 1.8s		WF:<br>FRC = 253±74 nm<br>Decorrelation = 297 nm<br>SIM:<br>FRC= 202±47 nm<br>Decorrelation= 117 nm
Live-cell TIRF imaging of cultured neurons expressing SkylanNS tagged tubulin	3a), ED5b)	405 nm: 0.727 W/cm^2<br>488 nm: 0.682 W/cm^2	10 Hz, 20s	eSRRF: M=4, R=5, S=2, VAR, 200 fr<br>SRRF: M=2, R=3, A=6, 200 fr	WF:<br>FRC =: 425±42 nm<br>Decorrelation = 339 nm,<br>SRRF:<br>FRC = 213±41 nm<br>Decorrelation: 132 nm,<br>eSRRF:

					FRC = 193±51 nm<br>Decorrelation: 125 nm
Nuclear pore complex in fixed isolated nuclear envelopes (Dataset from Heil et al.[24])	S4	5 kW/cm^2	200Hz	M=10,R=3,S=10, AVG, 2000 fr/20000fr	WF:<br>FRC = 410 nm<br>Decorrelation = 384 nm<br>eSRRF:<br>FRC (2000 fr)=44.4±2.5 nm,<br>Decorrelation (2000 fr)= 80 nm<br>Decorrelation (20000 fr)= 66 nm<br>SMLM:<br>FRC (20000 fr): 35.1±6.3 nm<br>Decorrelation (2000 fr) = 51 nm<br>Decorrelation (20000 fr) = 41 nm
Live-cell SDC of U2OS cells in PAM	ED8a)	4.6 W/cm^2*	10 Hz/10s	M=5, R=2, S=1, AVG, 100 frames	WF:<br>FRC=573.0±7.2 nm<br>Decorrelation=1030 nm<br>eSRRF:<br>FRC=197±34 nm<br>Decorrelation=260 nm
Two-color SDC of fixed spheroids (collagen I/actin)	ED8b)	4.6/2.6 W/cm^2*	10 Hz/10s	M=5, R= 2/3, S=1, AVG, 100 frames	WF:<br>FRC(Actin)= 569±59 nm<br>FRC(Collagen I)= 583±14 nm,<br>Decorrelation(Actin)= 624 nm<br>Decorrelation(Collagen I)= 620 nm)<br>eSRRF:<br>FRC(Actin)= 229±97 nm<br>FRC(Collagen I)= 130±36 nm,<br>Decorrelation(Actin)= 370 nm<br>Decorrelation(Collagen I)= 204 nm
In-vivo SDC of zebrafish	ED8c)	2.6 W/cm^2*	10 Hz/10s	M=5, R= 2, S=1, AVG, 150 frames	WF:<br>FRC(top)= 641±82 nm

					FRC(middle)= 573.9±5.7 nm<br>FRC(bottom)= 575.9±7.9 nm<br>Decorrelation(top)= 1026 nm<br>Decorrelation(middle)= 998 nm<br>Decorrelation(bottom)= 1006 nm<br>eSRRF:<br>FRC(top)= 194±37 nm<br>FRC(middle)= 393±23 nm<br>FRC(bottom)= 307±60 nm<br>Decorrelation(top)= 236 nm<br>Decorrelation(middle)= 215 nm<br>Decorrelation(bottom)= 263 nm
Live-cell SDC imaging of U2OS cells expressing SkylanS-ßActin	3e), M4	405 nm: 0.290 W/cm^2, 488 nm: 0.766 W/cm^2	2 Hz/12h 10min, 10 min intervals	M=5, R=2.5, S=1, AVG, 50 frames	WF:<br>FRC = 484 ± 53 nm<br>Decorrelation = 903 nm<br>eSRRF:<br>FRC = 151 ± 77 nm<br>Decorrelation = 435 nm<br>SRRF:<br>FRC = 215 ± 63 nm<br>Decorrelation = 475 nm

Supplementary Table 4: Laser intensities, imaging speed, total imaging time, eSRRF and SRRF parameters and resolution estimates (FRC and Decorrelation analysis) for the data sets included in this paper. *Intensity at the coverslip surface.

Method	Reference	Basic principles	Type	Implemen-tation	Volume (µm^3)	Speed intrinsic/SR (Vol/sec)
3D eSRRF	this paper	MFM + eSRRF	Fluctuation analysis on specialized hardware	Fiji-Plugin	20x20x3.6	50 Hz/~1 Hz (live-cell)
3D SOFI	Geissbuehler *et al.* 2014[25]/Descloux *et al.* 2018[26]	MFM+SOFI	Fluctuation analysis on specialized hardware	Matlab library	65x65x3.5/ 50x50x2.5	40 Hz/~1Hz (live-cell)
RFBA	Chen *et al.* 2020[14]	Bessel light sheet + SRRF guided 3B	Fluctuation analysis on specialized hardware	Matlab/C++	216×13.8×14	0.42 Hz/0.014 Hz (live-cell)
IDDR-SPIM	Zhao *et al.* 2022[27]	Double-ring -modulated SPIM + deep-learning SRM	Deep-learning based super-resolution on specialized hardware	Python	10x220x13	~17 Hz/~17 Hz (live-cell)
RIM	Mangeat *et al.* 2021[28]	Speckled illumination + SOFI/SIM reconstruction	Fluctuation analysis on specialized hardware	C++	30x30x0.8	~20 Hz/0.2 Hz (live-cell)
3DpRESOLFT	Bodén *et al.* 2021[29]	Paralelized stimulated emission depletion with RSFPs and 3D interference patterns	Specialized hardware	-	40x40x1.6	-/0.1 Hz

Supplementary Table 5: Live-cell 3D super-resolution methods. Abbreviations: RFBA - radial fluctuation Bayesian analysis, 3B - Bayesian analysis of blinking and bleaching, SPIM - selective plane illumination microscopy, RIM - random illumination microscopy, 3DpRESOLFT - 3D, parallelized, reversible, saturable/switchable optical fluorescence transition microscopy, RSFPs - reversibly switchable fluorescent proteins

Supplementary Figures

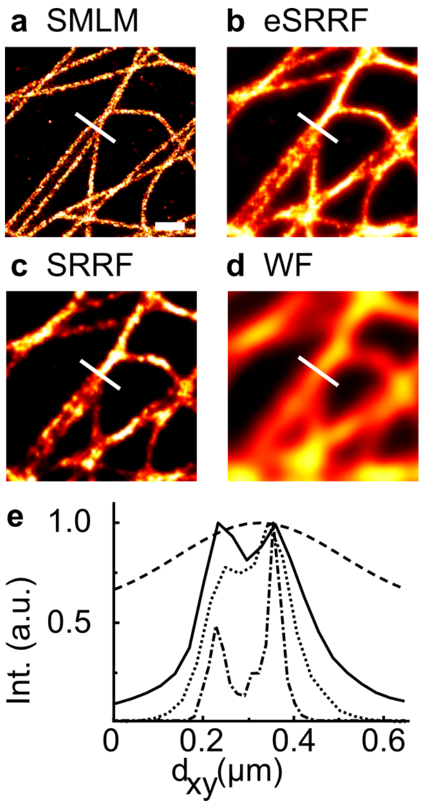

Supplementary Fig. 1: Resolution improvement of eSRRF vs. SRRF. Image sections of the data set presented in Fig. 1 after **a** SMLM image reconstruction, **b** eSRRF processing, **c** SRRF processing and **d** as WF data. The white line indicates the position of the line profiles. **e** Intensity profiles allow to distinguish two filaments in the SMLM reconstruction (dash-dotted line) which can also be resolved with eSRRF (solid line) but the presence of a second filament is unclear in the case of SRRF processing (dotted line) and for the WF data (dashed line). Scale bar: 500 nm.

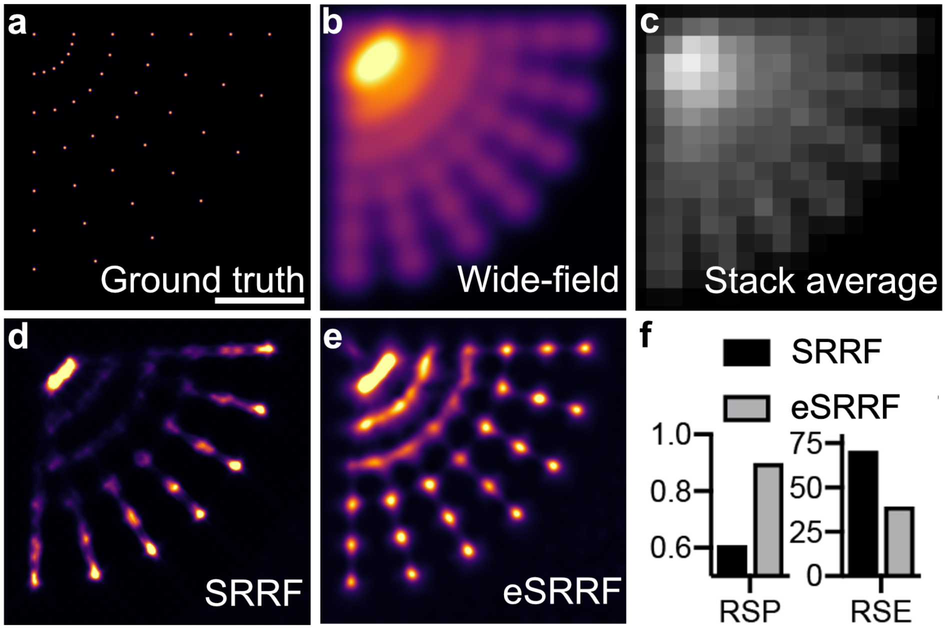

Supplementary Fig. 2: Performance improvement of eSRRF on simulated data. a Simulated ground truth indicating the positions of individual molecules placed on concentric rings with radii increasing by 220 nm steps. On each ring the molecules are separated by 57.5, 115, 173, 230, 288 and 345 nm, respectively. **b** Interpolated wide-field image. **c**, Average of all simulated raw frames **d**, SRRF image (R = 1, A = 6, 500 fr, TRAC2) **e,** eSRRF image (R = 1, S = 1, 500 fr, TAC2). **f** Quantitative comparisons of SRRF and eSRRF based on RSP and RSE obtained from SQUIRREL. Artifacts like the linearity loss and and over sharpening as they are observed in **d** are significantly reduced with **e**, eSRRF processing, Scale bar: 500 nm.

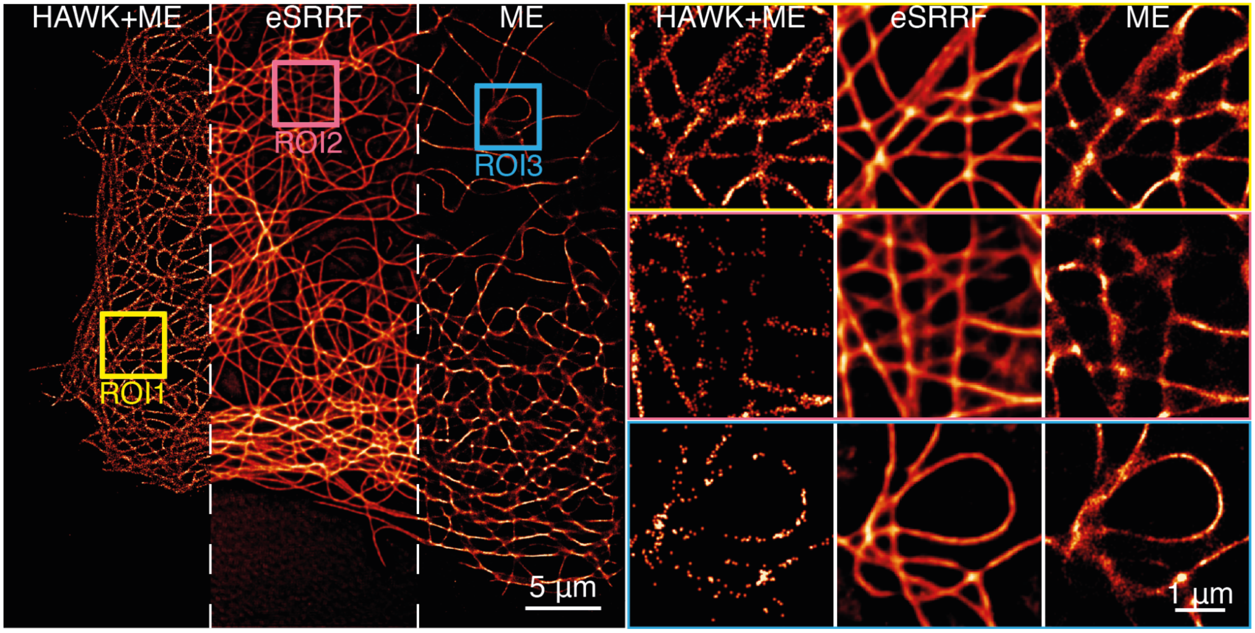

Supplementary Fig. 3: Comparison of eSRRF, HAWK+ME and ME. The Maximum Likelihood Estimation multi-emitter fitting (ME) was performed using ThunderSTORM[30]. Data shown corresponds to a DNA-PAINT acquisition of immunolabeled microtubules in fixed COS7 cells under TIRF illumination.

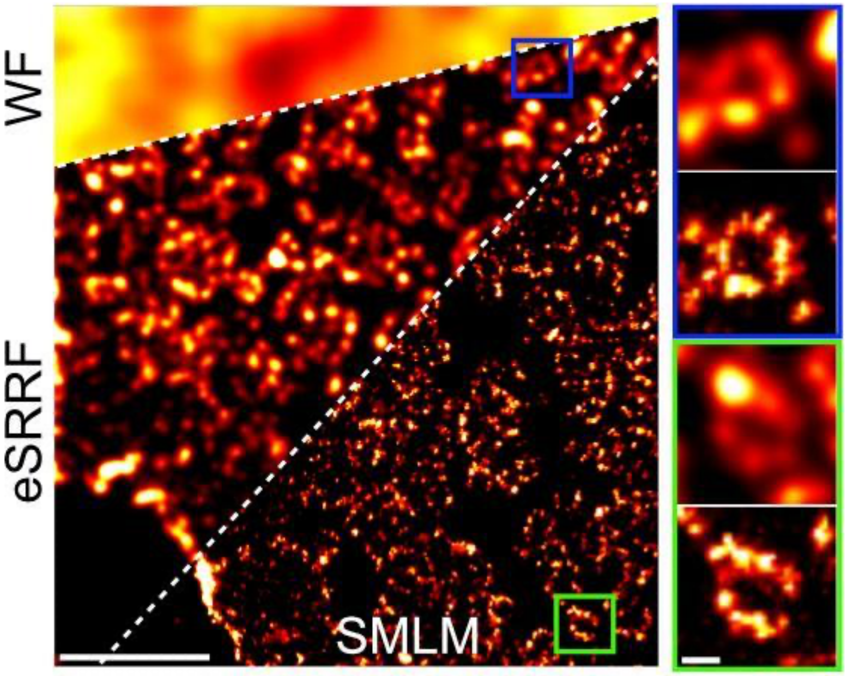

Supplementary Fig. 4: eSRRF allows a fast preview of SMLM dataset reconstruction. Widefield imaging (top left) of the nuclear pore complex. Fast eSRRF reconstruction preview (first 2000 frames shown) reveals the open ring structure (left panel middle area and insets top panels). While fast eSRRF can resolve the central pore which has a diameter of about 140 nm, the full 8-element ring with only 40 nm gaps is only resolved by single-molecule localization analysis of the full 20 000 frames image stack (left panel bottom area and insets lower panels). The FRC resolution is 44.4±2.5 nm, and 35.1±6.3 for eSRRF and SMLM, respectively) (Dataset from Heil et al.[24]), left panel: scale bar 1 µm, insets on the right: scale bar 100 nm, FRC shown as mean ± standard deviation.

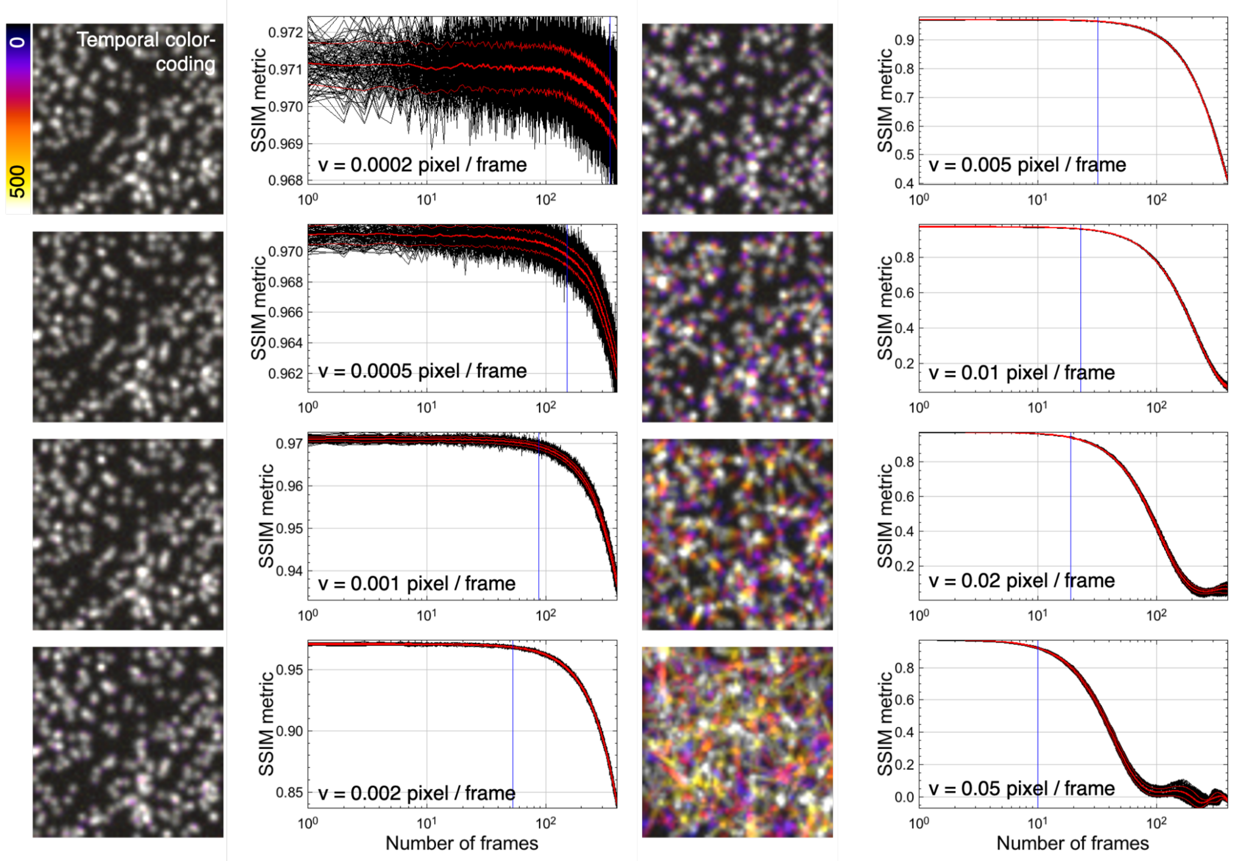

Supplementary Fig. 5: tSSIM analysis of simulated moving particles. Color coded projections of simulated image stacks displaying particles diffusing with various speeds *v* and the resulting SSIM metric progression over time. The tSSIM metric shows sensitivity as a function of particle displacement per frame. This can be used to estimate the size of the optimal time window for eSRRF processing to avoid movement artifacts.

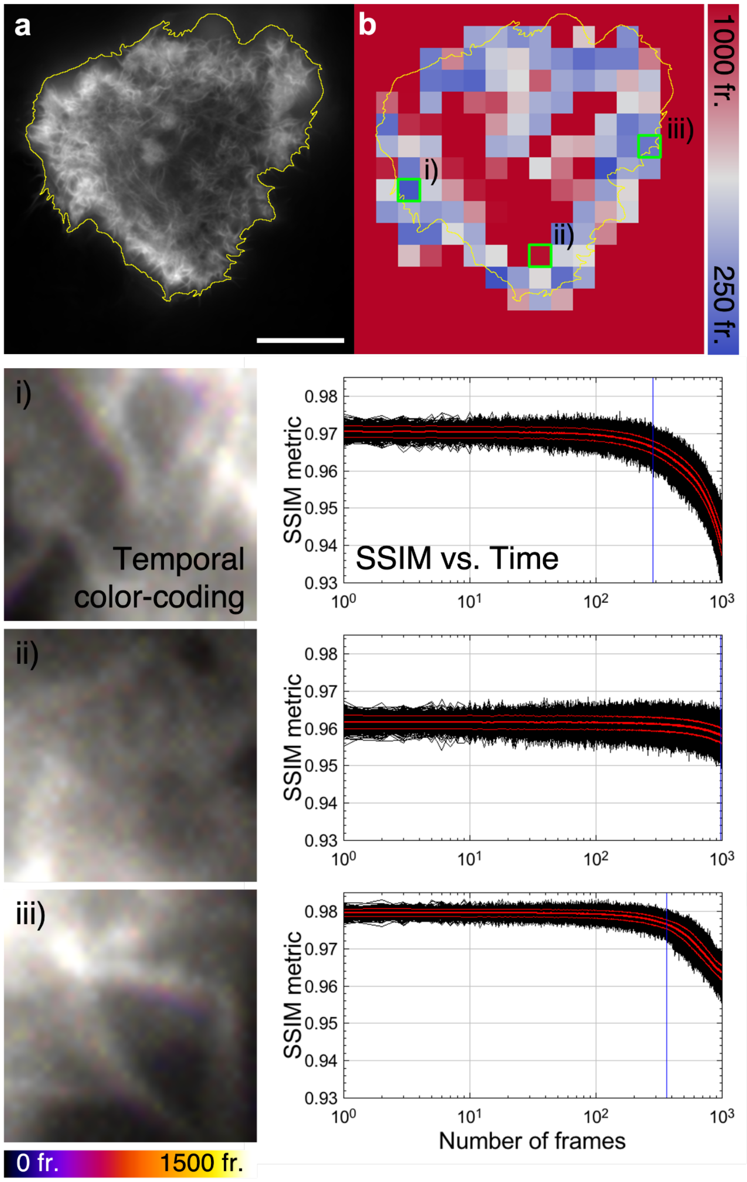

Supplementary Fig. 6: tSSIM analysis performed over small image patches detects local dynamics of actin rearrangement in COS-7 cells. a The actin network in live COS-7 cells expressing the marker GFP-UtrCH was acquired at 33 fps. **b** The tSSIM metric estimates the time range of motion within individual subsections of a). The different subsections highlighted in green display the color-coded projection of regions with i) fast, ii) slow, and iii) moderate speeds as is also reflected by the corresponding progression of the SSIM metric over time. Scale bar 20 μm.

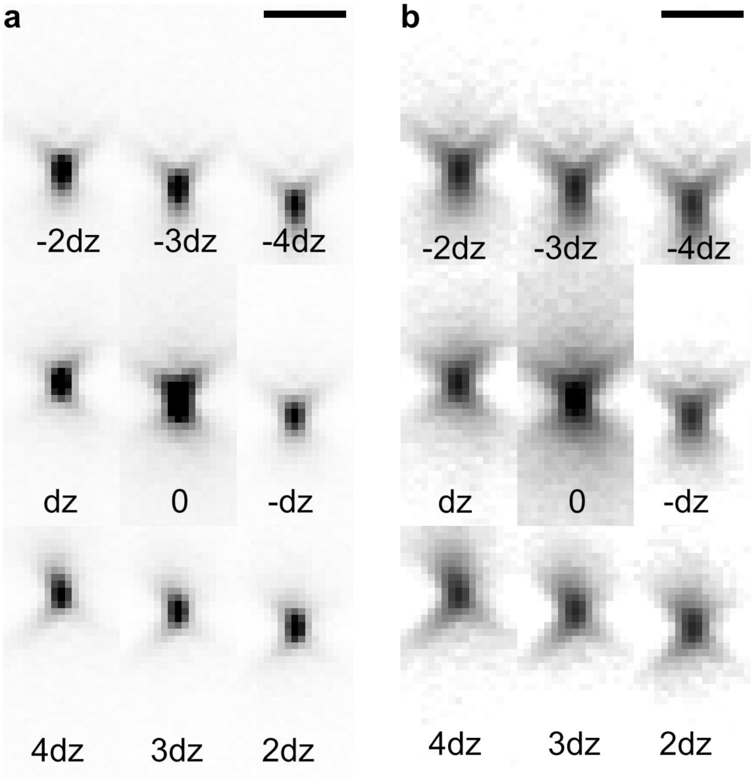

Supplementary Fig. 7: 3D PSF in the nine focus planes of the MFM displays only minor aberrations and good radial symmetry. x-z view of the PSF mapped with the bead calibration dataset displayed in **a** linear and **b** logarithmic brightness scale ($FWHM_X$=431±19 nm, $FWHM_z$=704±45 nm, mean ± standard deviation)**.** The focus offset dz between each focal plane is 390 nm. Scale bars 2 µm.

Supplementary Movies

Supplementary Movie 1: Automated reconstruction parameter search tool implemented in eSRRF. 200 frames of the live-cell TIRF imaging dataset of COS-7 cells expressing Lyn kinase – SkylanS were analyzed with eSRRF covering the Radius *R* and Sensitivity *S* parameter space defined by R_{start} =1, step size=0.5, number of steps=5 and S_{start} =1, step size=1, number of steps=5. The eSRRF reconstruction for each parameter combination is presented on the left, while the corresponding image resolution and fidelity is marked with a yellow square in the respective FRC and RSP maps. At a low R values pixel artifacts are evident, while at higher R values and low S values no high resolution is achieved. If both, R and S values, are high the reconstruction displays a high degree of nonlinearity. The compromise between resolution and fidelity is represented in the QnR map which displays a maximum at the parameter combination R=2 and S=4 (marked in red).

Supplementary Movie 2: Lattice-light sheet imaging of ER in live Jurkat T-cells enhanced by eSRRF. Slice-by-slice processing of the data set allows the reconstruction of a volumetric view (79 x 55 x 35 μm^3) of the ER network in live Jurkat T-cells at a rate of 7.6 mHz.

Supplementary Movie 3: Live-cell HiLO-TIRF of COS-7 cells expressing PrSS-mEmerald-KDEL marking the ER lumen. WF and eSRRF reconstruction of COS-7 cells expressing a luminal ER marker allows live-cell super-resolution imaging (FRC resolution HiLO/eSRRF (mean ± standard deviation): 254±11/143±56 nm) at a sampling rate of 1 Hz. Rolling window analysis allows to speed up temporal sampling to 10 Hz, FRC shown as mean ± standard deviation.

Supplementary Movie 4: Live-cell imaging of actin dynamics in U2OS cells expressing SkylanS-ßActin. The dynamic actin rearrangement in U2OS cells transiently expressing SkylanS-ßActin is visualized over a time course of 12 hours by acquiring substacks of 50 frames to generate a super-resolved eSRRF reconstruction at 10 min intervals. eSRRF processing allows to improve the resolution significantly (FRC resolution est. SDC/eSRRF: 484 ± 53 nm/151 ± 77 nm). SRRF processing does not achieve the same level of resolution improvement (FRC resolution est.: 215 ± 63 nm). Scale bar 10 µm, FRC shown as mean ± standard deviation.

Supplementary Movie 5: Live-cell 3D eSRRF of mitochondria dynamics with MFM. Live-cell volumetric imaging of U2OS cells expressing TOM20-Halo, loaded with JF549 with MFM (top left: single xy plane, middle left: single xz plane, bottom left: 3D rendering, FRC resolution estimate: 317 ± 22 nm) of a 20 x 20 x 3.6 µm3 observed over 3 min 18 sec allows to reconstruct a super-resolved in 3D view of them mitochondria dynamics with eSRRF processing ((top right: single xy plane, middle right: single xz plane, bottom right: 3D rendering, right, FRC resolution est., xy: 124 ± 60 nm/xz: 222 ± 26 nm) ~1Hz, scale bar 3 µm, FRC shown as mean ± standard deviation.

References

1. Marsh, R. J. *et al.* Artifact-free high-density localization microscopy analysis. *Nat. Methods* **15**, 689–692 (2018).
2. Culley, S. *et al.* Quantitative mapping and minimization of super-resolution optical imaging artifacts. *Nat. Methods* **15**, 263–266 (2018).
3. Parthasarathy, R. Rapid, accurate particle tracking by calculation of radial symmetry centers. *Nat. Methods* **9**, 724–726 (2012).
4. Roberts, L. Machine perception of 3-D solids, optical and electro-optical information processing. (1965).
5. Mendel, J. M. Tutorial on higher-order statistics (spectra) in signal processing and system theory: theoretical results and some applications. *Proc. IEEE* **79**, 278–305 (1991).
6. Abrahamsson, S. *et al.* Fast multicolor 3D imaging using aberration-corrected multifocus microscopy. *Nat. Methods* **10**, 60–63 (2013).
7. Hajj, B. *et al.* Whole-cell, multicolor superresolution imaging using volumetric multifocus microscopy. *Proc. Natl. Acad. Sci.* **111**, 17480–17485 (2014).
8. Hajj, B., Oudjedi, L., Fiche, J.-B., Dahan, M. & Nollmann, M. Highly efficient multicolor multifocus microscopy by optimal design of diffraction binary gratings. *Sci. Rep.* **7**, 5284 (2017).
9. Laine, R. F. *et al.* NanoJ: a high-performance open-source super-resolution microscopy toolbox. *J. Phys. Appl. Phys.* **52**, 163001 (2019).
10. Royer, L. A. *et al.* ClearVolume: open-source live 3D visualization for light-sheet microscopy. *Nat. Methods* **12**, 480–481 (2015).
11. Gustafsson, N. *et al.* Fast live-cell conventional fluorophore nanoscopy with ImageJ through super-resolution radial fluctuations. *Nat. Commun.* **7**, 12471 (2016).
12. Agarwal, K. & Macháň, R. Multiple signal classification algorithm for super-resolution

fluorescence microscopy. *Nat. Commun.* **7**, 13752 (2016).

13. Cox, S. *et al.* Bayesian localization microscopy reveals nanoscale podosome dynamics. *Nat. Methods* **9**, 195–200 (2012).
14. Chen, R. *et al.* Efficient super-resolution volumetric imaging by radial fluctuation Bayesian analysis light-sheet microscopy. *J. Biophotonics* **13**, (2020).
15. Dertinger, T., Colyer, R., Iyer, G., Weiss, S. & Enderlein, J. Fast, background-free, 3D super-resolution optical fluctuation imaging (SOFI). *Proc. Natl. Acad. Sci.* **106**, 22287–22292 (2009).
16. Zhao, W., Liu, J. & Li, H. Ultrafast super-resolution imaging via auto-correlation two-step deconvolution. in *Ultrafast Nonlinear Imaging and Spectroscopy VIII* vol. 11497 42–49 (SPIE, 2020).
17. Zhao, W. *et al.* Enhancing detectable fluorescence fluctuation for high-throughput and four-dimensional live-cell super-resolution imaging. Preprint at https://doi.org/10.1101/2022.12.12.520072 (2022).
18. Zeng, Z., Ma, J. & Xu, C. Cross-cumulant enhanced radiality nanoscopy for multicolor superresolution subcellular imaging. *Photonics Res.* **8**, 893 (2020).
19. Zeng, Z., Ma, J., Xi, P. & Xu, C. Joint tagging assisted fluctuation nanoscopy enables fast high-density super-resolution imaging. *J. Biophotonics* **11**, e201800020 (2018).
20. Gong, X. *et al.* Achieving increased resolution and reconstructed image quality with gradient variance modified super-resolution radial fluctuations. Preprint at https://doi.org/10.21203/rs.3.rs-608461/v1 (2021).
21. Shaib, A. H. *et al.* Expansion microscopy at one nanometer resolution. Preprint at https://doi.org/10.1101/2022.08.03.502284 (2022).
22. Kylies, D. *et al.* Expansion-enhanced super-resolution radial fluctuations enable nanoscale molecular profiling of pathology specimens. *Nat. Nanotechnol.* **18**, 336–342 (2023).
23. Moeyaert, B. & Dedecker, P. A comprehensive dataset of image sequences covering 20

fluorescent protein labels and 12 imaging conditions for use in super-resolution imaging. *Data Brief* **29**, 105273 (2020).

24. Heil, H. S. *et al.* Sharpening emitter localization in front of a tuned mirror. *Light Sci. Appl.* **7**, 99 (2018).
25. Geissbuehler, S. *et al.* Live-cell multiplane three-dimensional super-resolution optical fluctuation imaging. *Nat. Commun.* **5**, 5830 (2014).
26. Descloux, A. *et al.* Combined multi-plane phase retrieval and super-resolution optical fluctuation imaging for 4D cell microscopy. *Nat. Photonics* **12**, 165–172 (2018).
27. Zhao, Y. *et al.* Isotropic super-resolution light-sheet microscopy of dynamic intracellular structures at subsecond timescales. *Nat. Methods* **19**, 359–369 (2022).
28. Mangeat, T. *et al.* Super-resolved live-cell imaging using random illumination microscopy. *Cell Rep. Methods* **1**, 100009 (2021).
29. Bodén, A. *et al.* Volumetric live cell imaging with three-dimensional parallelized RESOLFT microscopy. *Nat. Biotechnol.* **39**, 609–618 (2021).
30. Ovesný, M., Křížek, P., Borkovec, J., Švindrych, Z. & Hagen, G. M. ThunderSTORM: a comprehensive ImageJ plug-in for PALM and STORM data analysis and super-resolution imaging. *Bioinformatics* **30**, 2389–2390 (2014).
