# Supplementary material for: High-fidelity 3D live-cell nanoscopy through data-driven enhanced super-resolution radial fluctuation
Source: Nat Methods. 2023 Nov 13;20(12):1949–56. doi: 10.1038/s41592-023-02057-w (PMC10703683; doi:10.1038/s41592-023-02057-w)
Supplement: Supplementary file 2 — Reporting Summary [file 41592_2023_2057_MOESM2_ESM.pdf]

nature research

Corresponding author(s): Bassam Hajj, Christophe Leterrier, Ricardo Henriques

Last updated by author(s): Sep 4, 2023

Reporting Summary

Nature Research wishes to improve the reproducibility of the work that we publish. This form provides structure for consistency and transparency in reporting. For further information on Nature Research policies, see our Editorial Policies and the Editorial Policy Checklist.

Statistics

For all statistical analyses, confirm that the following items are present in the figure legend, table legend, main text, or Methods section.

n/a	Confirmed	
☐	☒	The exact sample size (*n*) for each experimental group/condition, given as a discrete number and unit of measurement
☒	☐	A statement on whether measurements were taken from distinct samples or whether the same sample was measured repeatedly
☒	☐	The statistical test(s) used AND whether they are one- or two-sided<br>*Only common tests should be described solely by name; describe more complex techniques in the Methods section.*
☒	☐	A description of all covariates tested
☒	☐	A description of any assumptions or corrections, such as tests of normality and adjustment for multiple comparisons
☐	☒	A full description of the statistical parameters including central tendency (e.g. means) or other basic estimates (e.g. regression coefficient) AND variation (e.g. standard deviation) or associated estimates of uncertainty (e.g. confidence intervals)
☒	☐	For null hypothesis testing, the test statistic (e.g. *F*, *t*, *r*) with confidence intervals, effect sizes, degrees of freedom and *P* value noted<br>*Give P values as exact values whenever suitable.*
☒	☐	For Bayesian analysis, information on the choice of priors and Markov chain Monte Carlo settings
☒	☐	For hierarchical and complex designs, identification of the appropriate level for tests and full reporting of outcomes
☐	☒	Estimates of effect sizes (e.g. Cohen's *d*, Pearson's *r*), indicating how they were calculated

Our web collection on statistics for biologists contains articles on many of the points above.

Software and code

Policy information about availability of computer code

Data collection	Commercial: Acquisition with Nikon-NIS Elements 5.30.02/5.30.05 (Nikon), SlideBook V6 (Intelligent Imaging Innovations, Inc.), ZEN Black Version 11.0.2.190 (Carl Zeiss)<br>Open Source: Fiji/ImageJ 1.45f (plugins: NanoJ-eSRRF)<br>Custom code: Diffusing particle simlation: Python script available as GoogleCoLabs Jupyter notebook on GitHub: https://github.com/HenriquesLab/NanoJ-eSRRF.
Data analysis	Commercial: Huygens Professional version 21.10 (Scientific Volume Imaging, The Netherlands, http.//svi.nl)<br>Open Source: Fiji/ImageJ 1.45f (plugins: NanoJ-eSRRF, ThunderSTORM, NanoJ-SQUIRREL, LLSM, HAWK, DeconvolutionLab2), devbio-Napari (https://github.com/haesleinhuepf/devbio-napari)

For manuscripts utilizing custom algorithms or software that are central to the research but not yet described in published literature, software must be made available to editors and reviewers. We strongly encourage code deposition in a community repository (e.g. GitHub). See the Nature Research guidelines for submitting code & software for further information.

Data

Policy information about availability of data

All manuscripts must include a data availability statement. This statement should provide the following information, where applicable:

- Accession codes, unique identifiers, or web links for publicly available datasets
- A list of figures that have associated raw data
- A description of any restrictions on data availability

Datasets are available on Zenodo (https://doi.org/10.5281/zenodo.6466472).

April 2020

Field-specific reporting

Please select the one below that is the best fit for your research. If you are not sure, read the appropriate sections before making your selection.

☒ Life sciences ☐ Behavioural & social sciences ☐ Ecological, evolutionary & environmental sciences

For a reference copy of the document with all sections, see nature.com/documents/nr-reporting-summary-flat.pdf

Life sciences study design

All studies must disclose on these points even when the disclosure is negative.

Sample size	No sample-size calculation was performed as the manuscript reports the demonstration of an image reconstruction method, we chose the sample size that can validate reproducibility of our technique. The manuscript draws no biological conclusions, and does not examine or compare different biological conditions. This is not a life science study with comparative analyses of a certain sample size.
Data exclusions	No data was excluded from the analysis
Replication	All attempts of replication were successful and results supported by simulations. All experiments were repeated three or more times with similar results.
Randomization	Randomization is not relevant to this study as this is not a life science study with comparative analyses of biological situations, but a validation of the performance of an algorithm supported by a data driven parameter optimization to avoid user bias.
Blinding	No blinding was performed. Blinding is not relevant because there is no comparison of different biological situations performed in this work.

Reporting for specific materials, systems and methods

We require information from authors about some types of materials, experimental systems and methods used in many studies. Here, indicate whether each material, system or method listed is relevant to your study. If you are not sure if a list item applies to your research, read the appropriate section before selecting a response.

Materials & experimental systems

n/a	Involved in the study	
☐	☒	Antibodies
☐	☒	Eukaryotic cell lines
☒	☐	Palaeontology and archaeology
☐	☒	Animals and other organisms
☒	☐	Human research participants
☒	☐	Clinical data
☒	☐	Dual use research of concern

Methods

n/a	Involved in the study	
☒	☐	ChIP-seq
☒	☐	Flow cytometry
☒	☐	MRI-based neuroimaging

Antibodies

Antibodies used	anti-α-tubulin mouse monoclonal IgG1 antibodies (DM1A (T6199, Sigma) and B-5–1-2 (T5168, Sigma)<br>goat anti-mouse antibody conjugated to a DNA sequence (P1 docking strand, U10001, Ultivue kit, discontinued)
Validation	DM1A: The isotype is determined by a double diffusion immunoassay using Mouse Monoclonal Antibody Isotyping Reagents, Product Number ISO2. Anti-a-Tubulin antibody, Mouse monoclonal recognizes an epitope located at the C-terminal end of the a-tubulin isoform (amino acids 426-430) in a variety of organisms (e.g., human, bovine, mouse, and chicken).<br>References:<br>1. Blose, S.H., et al., J. Cell Biol., 98, 847-858 (1984).<br>2. Breitling, F., and Little, M., J. Molec. Biol., 189, 367-370 (1986).<br>3. Wolff, A., et al., Biol. Cell, 63, 319-326 (1988).<br>4. Serrano, L., et al., Anal. Biochem., 159, 253-259 (1986).<br>B-5–1-2: The isotype is determined by a double diffusion immunoassay using Mouse Monoclonal Antibody Isotyping Reagents, Catalog Number ISO2. Monoclonal Anti-a-Tubulin recognizes an epitope located in the C-terminal end of the a-tubulin isoform in a variety of organisms (e.g., human, sea urchin,Chlamydomonas).<br>1. Piperno, G., et al., J. Cell Biol., 104, 289 (1987).<br>2. LeDizet, M., and Piperno, G., Meth. Enzymol., 196,264 (1991).<br>3. LeDizet, M., and Piperno, G.,

Eukaryotic cell lines

Policy information about cell lines

Cell line source(s)	COS-7 cells (ATCC CRL-1651), HeLa cells (ATCC CRM-CCL-2), Jurkat (Cellbank Australia Jurkat-ILA1), U2OS (ATCC HTB-96 & Leibniz Institute DSMZ-German Collection of Microorganisms and Cell Cultures, Braunschweig DE, ACC 785), MCF10 DCIS.COM (DCIS.COM)
Authentication	Cell lines from ATCC, DSMZ, DCIS.COM or Cellbank Australia used at low passage numbers and authenticated with a morphology check under the light microscope.
Mycoplasma contamination	All cell lines were tested negative for mycoplasma contamination on a regular basis.
Commonly misidentified lines (See ICLAC register)	No commonly misidentified cell lines were used in the study.

Animals and other organisms

Policy information about studies involving animals; ARRIVE guidelines recommended for reporting animal research

Laboratory animals	Housing and experimentation of Zebrafish (Danio rerio, strain Tg(KDR:mcherryCAAX, age 2 days post fertilization, sex not not determinable) were performed under license MMM/465/712-93 (issued by the Ministry of Agriculture and Forestry, Finland). Rat neuronal cultures were prepared from Wistar rat day 18 embryos of both sexes in agreement with the guidelines established by the European Animal Care and Use Committee (86/609/CEE) and was approved by the Aix-Marseille University ethics committee 14 (France, agreement G13O555)
Wild animals	The study did not involve wild animals.
Field-collected samples	The study did not involve field-collected samples.
Ethics oversight	Zebrafish handling was performed under license MMM/465/712-93 issued by the Ministry of Agriculture and Forestry, Finland. Rat neuronal culturing was performed under license G13O555 issued by the Aix-Marseille University ethics committee 14 (France).

Note that full information on the approval of the study protocol must also be provided in the manuscript.
